# Swietenolide diacetate from the seeds of *Swietenia macrophylla*

**DOI:** 10.1107/S1600536810017733

**Published:** 2010-05-22

**Authors:** Bey Hing Goh, Habsah Abdul Kadir, Sri Nurestri Abdul Malek, Seik Weng Ng

**Affiliations:** a Institute of Biological Sciences, University of Malaya, 50603 Kuala Lumpur, Malaysia; b Department of Chemistry, University of Malaya, 50603 Kuala Lumpur, Malaysia

## Abstract

The title compound, C_31_H_38_O_10_ [systematic name: (α*R*,4*R*,4a*R*,6a*S*,7*R*,8*S*,10*R*,11*S*)-methyl α,10-di­acet­oxy-4-(3-furyl)-4a,7,9,9-tetra­methyl-2,13-dioxo-1,4,4a,5,6,6a,7,8,9,10,11,12-dodeca­hydro-7,11-methano-2*H*-cyclo­octa­[*f*][2]benzo­pyran-8-acetate], was isolated from the seeds of *Swietenia macrophylla*. The mol­ecule contains four six-membered rings connected together in the shape of a bowl; one of the inner rings adopts a twisted chair conformation owing to the carbon–carbon double bond. The furyl substitutent is connected to an outer ring, and it points away from the bowl cavity.

## Related literature

For the isolation, spectroscopic characterization and absolute structure of the title compound, see: Chan *et al.* (1976 ▶); Connolly & Labbe (1980 ▶); Connolly *et al.* (1965 ▶); Govindachari *et al.* (1999 ▶); Kadota, Marpaung *et al.* (1990 ▶); Kadota, Yanagawa *et al.* (1990 ▶); Mootoo *et al.* (1999 ▶); Narender *et al.* (2008 ▶); Schefer *et al.* (2006 ▶); Taylor & Taylor (1983 ▶); Yuan *et al.* (2010 ▶).
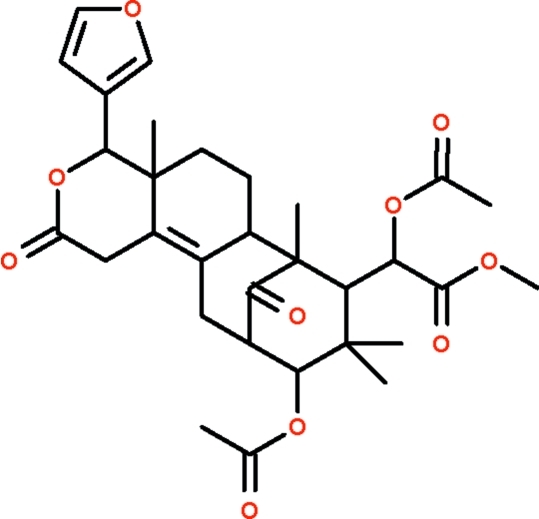


## Experimental

### 

#### Crystal data

C_31_H_38_O_10_
*M* *_r_* = 570.61
Orthorhombic, 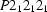

*a* = 12.5889 (11) Å
*b* = 13.7109 (12) Å
*c* = 17.0045 (14) Å
*V* = 2935.1 (4) Å^3^
*Z* = 4
Mo *K*α radiation
μ = 0.10 mm^−1^
*T* = 293 K
0.35 × 0.15 × 0.10 mm

#### Data collection

Bruker SMART APEX diffractometer
28065 measured reflections
3771 independent reflections
2491 reflections with *I* > 2σ(*I*)
*R*_int_ = 0.077

#### Refinement

*R*[*F* ^2^ > 2σ(*F* ^2^)] = 0.043
*wR*(*F* ^2^) = 0.127
*S* = 1.02
3771 reflections
377 parameters
H-atom parameters constrained
Δρ_max_ = 0.16 e Å^−3^
Δρ_min_ = −0.13 e Å^−3^

### 

Data collection: *APEX2* (Bruker, 2009 ▶); cell refinement: *SAINT* (Bruker, 2009 ▶); data reduction: *SAINT*; program(s) used to solve structure: *SHELXS97* (Sheldrick, 2008 ▶); program(s) used to refine structure: *SHELXL97* (Sheldrick, 2008 ▶); molecular graphics: *X-SEED* (Barbour, 2001 ▶); software used to prepare material for publication: *publCIF* (Westrip, 2010 ▶).

## Supplementary Material

Crystal structure: contains datablocks global, I. DOI: 10.1107/S1600536810017733/bt5263sup1.cif

Structure factors: contains datablocks I. DOI: 10.1107/S1600536810017733/bt5263Isup2.hkl

Additional supplementary materials:  crystallographic information; 3D view; checkCIF report

## Figures and Tables

**Fig. 1.**
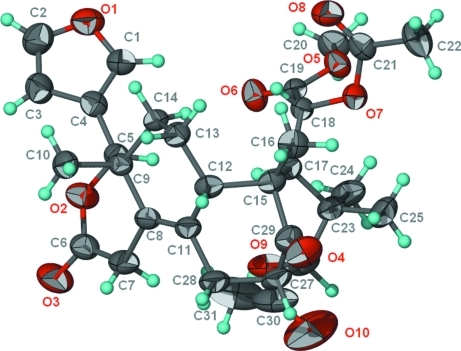
Anisotropic displacement ellipsoid plot (Barbour, 2001) of C31H38O10 at the 50% probability level; hydrogen atoms are drawn as spheres of arbitrary radius.

## supplementary crystallographic information

### Comment

*Sweietenia macrophylla* is a large mahogany tree growing in the rainforests of Malaysia. The extracts of the seeds contain flavonoids, saponins and alkaloids that are commecialized in local herbal products. The isolation of the title compound (Scheme I, Fig. 1) has been reported a long time ago. The present crystal structure analysis confirms the spectroscopic structure determination.

### Experimental

Swietenolide diacetate was isloated from the seeds of *Swietenia macrophylla *by using a reported procedure (Chan *et al.*, 1976).

The finely ground seeds (600 g) were soaked in ethanol at room temperature for three days. The mixture was filtered and the sovlent evaporated to give a dark yellow crude material (70 g). A portion (40 g) was successively extracted with *n*-hexane, ethyl acetate and water to give an *n*-hexane-insoluble residue. The residue was partitioned between ethyl acetate-water (1:1) to give an ethyl acetate-soluble fraction (30 g, 80%).

This fraction (3 g) was subjected to column chromatography on silica gel (70-230 mesh, 300 g), with initial elution by *n*-hexane, followed by increasing proportions of chloroform. Eleven fractions were obtained. The fourth fraction (2 g) was further subjected to column chromatography (70-230 mesh,200 g), initially eluting with *n*-hexane and later with acetone to give twelve fractions.

The eighth fraction (600 mg) was dissolved in methanol and kept in a refrigerator. A white solid was obtained after two days, and a second crop was obtained after another two days. Recrystallization of the first crop from chloroform yielded colorless crystals of the title compound (yield 15 mg).

### Refinement

Carbon-bound H-atoms were placed in calculated positions (C—H 0.93 to 0.98 Å) and were included in the refinement in the riding model approximation, with *U*(H) set to 1.2 to 1.5*U*_eq_(C). The Flack parameter was fixed to be zero. 2976 Friedel pairs were merged.

### Crystal data

**Table:**

C_31_H_38_O_10_	*F*(000) = 1216
*M**_r_* = 570.61	*D*_x_ = 1.291 Mg m^−^^3^
Orthorhombic, *P*2_1_2_1_2_1_	Mo *K*α radiation, λ = 0.71073 Å
Hall symbol: P 2ac 2ab	Cell parameters from 4600 reflections
*a* = 12.5889 (11) Å	θ = 2.2–21.7°
*b* = 13.7109 (12) Å	µ = 0.10 mm^−^^1^
*c* = 17.0045 (14) Å	*T* = 293 K
*V* = 2935.1 (4) Å^3^	Prism, colorless
*Z* = 4	0.35 × 0.15 × 0.10 mm

### Data collection

**Table:**

Bruker SMART APEX diffractometer	2491 reflections with *I* > 2σ(*I*)
Radiation source: fine-focus sealed tube	*R*_int_ = 0.077
graphite	θ_max_ = 27.5°, θ_min_ = 1.9°
ω scans	*h* = −14→16
28065 measured reflections	*k* = −17→17
3771 independent reflections	*l* = −21→22

### Refinement

**Table:**

Refinement on *F*^2^	Primary atom site location: structure-invariant direct methods
Least-squares matrix: full	Secondary atom site location: difference Fourier map
*R*[*F*^2^ > 2σ(*F*^2^)] = 0.043	Hydrogen site location: inferred from neighbouring sites
*wR*(*F*^2^) = 0.127	H-atom parameters constrained
*S* = 1.02	*w* = 1/[σ^2^(*F*_o_^2^) + (0.0642*P*)^2^ + 0.2454*P*] where *P* = (*F*_o_^2^ + 2*F*_c_^2^)/3
3771 reflections	(Δ/σ)_max_ = 0.001
377 parameters	Δρ_max_ = 0.16 e Å^−^^3^
0 restraints	Δρ_min_ = −0.13 e Å^−^^3^

### Fractional atomic coordinates and isotropic or equivalent isotropic displacement parameters (Å^2^)

**Table:**

	*x*	*y*	*z*	*U*_iso_*/*U*_eq_	
O1	0.6572 (2)	0.3236 (2)	−0.20580 (14)	0.0747 (7)	
O2	0.7345 (2)	0.35757 (17)	0.04692 (14)	0.0651 (6)	
O3	0.7606 (3)	0.4265 (2)	0.16075 (17)	0.0924 (9)	
O4	0.9749 (2)	−0.1523 (2)	0.17405 (16)	0.0828 (8)	
O5	0.55375 (19)	−0.11379 (15)	−0.08732 (13)	0.0571 (6)	
O6	0.61162 (19)	0.03581 (15)	−0.06055 (15)	0.0622 (6)	
O7	0.72148 (19)	−0.20206 (14)	−0.03800 (12)	0.0521 (5)	
O8	0.7631 (3)	−0.19051 (19)	−0.16536 (15)	0.0807 (8)	
O9	0.6687 (2)	0.0434 (2)	0.19007 (13)	0.0720 (7)	
O10	0.5727 (4)	0.0137 (4)	0.2958 (3)	0.169 (2)	
C1	0.6620 (3)	0.2682 (3)	−0.13924 (19)	0.0620 (9)	
H1	0.6269	0.2091	−0.1325	0.074*	
C2	0.7183 (4)	0.4023 (3)	−0.1918 (2)	0.0801 (12)	
H2	0.7296	0.4527	−0.2274	0.096*	
C3	0.7605 (4)	0.3985 (3)	−0.1201 (2)	0.0729 (11)	
H3	0.8053	0.4445	−0.0975	0.087*	
C4	0.7242 (3)	0.3105 (2)	−0.08472 (19)	0.0520 (8)	
C5	0.7473 (3)	0.2725 (2)	−0.00418 (18)	0.0491 (7)	
H5	0.6923	0.2248	0.0095	0.059*	
C6	0.7706 (3)	0.3546 (3)	0.1211 (2)	0.0622 (9)	
C7	0.8189 (4)	0.2635 (3)	0.1517 (2)	0.0704 (11)	
H7A	0.7672	0.2321	0.1856	0.084*	
H7B	0.8790	0.2814	0.1844	0.084*	
C8	0.8569 (3)	0.1887 (2)	0.09253 (17)	0.0486 (7)	
C9	0.8563 (2)	0.2247 (2)	0.00792 (16)	0.0431 (7)	
C10	0.9466 (3)	0.2977 (2)	−0.0049 (2)	0.0582 (8)	
H10A	1.0136	0.2651	0.0011	0.087*	
H10B	0.9412	0.3493	0.0330	0.087*	
H10C	0.9416	0.3244	−0.0570	0.087*	
C11	0.8891 (3)	0.0997 (2)	0.11439 (17)	0.0481 (7)	
C12	0.9384 (2)	0.0273 (2)	0.05864 (17)	0.0485 (7)	
H12	1.0107	0.0171	0.0784	0.058*	
C13	0.9526 (3)	0.0679 (2)	−0.02434 (17)	0.0510 (7)	
H13A	1.0207	0.1009	−0.0273	0.061*	
H13B	0.9543	0.0140	−0.0613	0.061*	
C14	0.8658 (2)	0.1386 (2)	−0.04903 (16)	0.0455 (7)	
H14A	0.7985	0.1043	−0.0512	0.055*	
H14B	0.8812	0.1630	−0.1013	0.055*	
C15	0.8823 (2)	−0.0758 (2)	0.06588 (17)	0.0490 (7)	
C16	0.9398 (3)	−0.1515 (3)	0.0156 (2)	0.0609 (9)	
H16A	0.9102	−0.2149	0.0254	0.091*	
H16B	1.0140	−0.1518	0.0287	0.091*	
H16C	0.9315	−0.1354	−0.0390	0.091*	
C17	0.7588 (2)	−0.0670 (2)	0.05172 (17)	0.0438 (7)	
H17	0.7446	0.0033	0.0537	0.053*	
C18	0.7268 (3)	−0.09720 (19)	−0.03206 (16)	0.0432 (7)	
H18	0.7836	−0.0755	−0.0673	0.052*	
C19	0.6247 (3)	−0.0503 (2)	−0.06065 (16)	0.0443 (7)	
C20	0.4543 (3)	−0.0743 (3)	−0.1149 (2)	0.0680 (10)	
H20A	0.4184	−0.1222	−0.1464	0.102*	
H20B	0.4677	−0.0172	−0.1460	0.102*	
H20C	0.4107	−0.0572	−0.0707	0.102*	
C21	0.7431 (3)	−0.2402 (2)	−0.1093 (2)	0.0565 (8)	
C22	0.7397 (4)	−0.3485 (2)	−0.1083 (3)	0.0834 (13)	
H22A	0.8030	−0.3738	−0.1322	0.125*	
H22B	0.6787	−0.3706	−0.1371	0.125*	
H22C	0.7351	−0.3709	−0.0549	0.125*	
C23	0.6916 (3)	−0.1102 (3)	0.12000 (18)	0.0590 (9)	
C24	0.5717 (3)	−0.1013 (3)	0.1041 (2)	0.0760 (11)	
H24A	0.5331	−0.1158	0.1513	0.114*	
H24B	0.5517	−0.1465	0.0636	0.114*	
H24C	0.5556	−0.0361	0.0874	0.114*	
C25	0.7149 (4)	−0.2193 (3)	0.1370 (2)	0.0815 (13)	
H25A	0.7901	−0.2286	0.1426	0.122*	
H25B	0.6893	−0.2584	0.0941	0.122*	
H25C	0.6797	−0.2385	0.1846	0.122*	
C26	0.7198 (3)	−0.0507 (3)	0.19481 (19)	0.0641 (9)	
H26	0.6901	−0.0852	0.2402	0.077*	
C27	0.8403 (3)	−0.0385 (3)	0.20878 (18)	0.0599 (9)	
H27	0.8563	−0.0606	0.2623	0.072*	
C28	0.8853 (3)	0.0659 (3)	0.19881 (18)	0.0620 (9)	
H28A	0.8418	0.1109	0.2288	0.074*	
H28B	0.9566	0.0679	0.2205	0.074*	
C29	0.9034 (3)	−0.0992 (3)	0.1525 (2)	0.0599 (9)	
C30	0.5932 (4)	0.0658 (5)	0.2431 (3)	0.0993 (16)	
C31	0.5422 (5)	0.1596 (5)	0.2259 (3)	0.135 (3)	
H31A	0.4720	0.1484	0.2058	0.203*	
H31B	0.5833	0.1943	0.1875	0.203*	
H31C	0.5379	0.1976	0.2733	0.203*	

### Atomic displacement parameters (Å^2^)

**Table:**

	*U*^11^	*U*^22^	*U*^33^	*U*^12^	*U*^13^	*U*^23^
O1	0.0871 (19)	0.0801 (18)	0.0570 (15)	0.0094 (16)	−0.0090 (14)	−0.0018 (13)
O2	0.0688 (15)	0.0690 (14)	0.0576 (14)	0.0173 (13)	−0.0014 (12)	−0.0152 (12)
O3	0.123 (2)	0.0782 (17)	0.0757 (18)	0.0140 (19)	0.0052 (17)	−0.0303 (15)
O4	0.0820 (18)	0.0923 (19)	0.0742 (17)	0.0214 (17)	−0.0234 (15)	0.0242 (16)
O5	0.0546 (13)	0.0524 (12)	0.0643 (14)	−0.0015 (11)	−0.0160 (12)	−0.0047 (10)
O6	0.0572 (14)	0.0449 (12)	0.0844 (16)	0.0046 (10)	−0.0142 (13)	0.0017 (11)
O7	0.0649 (14)	0.0394 (10)	0.0521 (12)	0.0058 (10)	−0.0030 (11)	0.0013 (9)
O8	0.119 (2)	0.0714 (15)	0.0516 (15)	0.0022 (17)	0.0051 (15)	−0.0070 (13)
O9	0.0713 (16)	0.103 (2)	0.0417 (12)	0.0149 (15)	0.0016 (12)	−0.0055 (12)
O10	0.176 (5)	0.226 (5)	0.103 (3)	0.026 (4)	0.082 (3)	0.017 (3)
C1	0.070 (2)	0.064 (2)	0.0519 (19)	−0.0011 (19)	−0.0007 (18)	−0.0028 (17)
C2	0.098 (3)	0.065 (2)	0.077 (3)	0.011 (2)	−0.005 (3)	0.014 (2)
C3	0.093 (3)	0.0507 (19)	0.075 (3)	0.004 (2)	−0.019 (2)	0.0056 (17)
C4	0.0514 (19)	0.0491 (17)	0.0555 (19)	0.0085 (15)	−0.0043 (16)	−0.0057 (14)
C5	0.0475 (19)	0.0506 (17)	0.0493 (17)	−0.0008 (15)	0.0021 (14)	−0.0059 (14)
C6	0.067 (2)	0.066 (2)	0.054 (2)	−0.0016 (19)	0.0086 (18)	−0.0122 (17)
C7	0.099 (3)	0.066 (2)	0.0456 (18)	0.001 (2)	0.0063 (19)	−0.0097 (16)
C8	0.0516 (18)	0.0564 (18)	0.0377 (15)	−0.0057 (15)	0.0005 (14)	−0.0047 (14)
C9	0.0406 (16)	0.0487 (16)	0.0400 (15)	−0.0035 (13)	0.0003 (13)	−0.0020 (13)
C10	0.0514 (19)	0.063 (2)	0.060 (2)	−0.0124 (17)	−0.0019 (17)	0.0026 (17)
C11	0.0483 (18)	0.0587 (18)	0.0374 (15)	−0.0050 (16)	−0.0054 (14)	0.0009 (13)
C12	0.0408 (16)	0.0629 (18)	0.0419 (15)	0.0054 (15)	−0.0063 (15)	0.0020 (15)
C13	0.0502 (18)	0.0547 (17)	0.0481 (17)	0.0027 (16)	0.0063 (15)	0.0004 (14)
C14	0.0504 (17)	0.0508 (16)	0.0353 (13)	−0.0019 (14)	0.0025 (14)	0.0018 (13)
C15	0.0468 (17)	0.0568 (17)	0.0435 (16)	0.0072 (15)	−0.0059 (14)	0.0059 (14)
C16	0.058 (2)	0.0603 (19)	0.064 (2)	0.0185 (18)	−0.0057 (18)	0.0014 (17)
C17	0.0451 (17)	0.0465 (15)	0.0397 (14)	0.0014 (14)	−0.0031 (13)	0.0044 (13)
C18	0.0523 (17)	0.0366 (14)	0.0407 (15)	0.0025 (14)	−0.0043 (14)	0.0012 (12)
C19	0.0543 (18)	0.0414 (16)	0.0372 (14)	−0.0011 (14)	−0.0023 (14)	0.0008 (12)
C20	0.055 (2)	0.080 (2)	0.069 (2)	0.002 (2)	−0.0195 (19)	−0.0008 (19)
C21	0.056 (2)	0.0523 (18)	0.061 (2)	0.0036 (17)	−0.0063 (17)	−0.0084 (16)
C22	0.092 (3)	0.052 (2)	0.106 (3)	0.009 (2)	0.005 (3)	−0.022 (2)
C23	0.064 (2)	0.070 (2)	0.0426 (17)	−0.0084 (18)	−0.0010 (16)	0.0080 (15)
C24	0.059 (2)	0.116 (3)	0.053 (2)	−0.016 (2)	0.0049 (18)	0.010 (2)
C25	0.096 (3)	0.081 (3)	0.067 (2)	−0.022 (2)	−0.007 (2)	0.029 (2)
C26	0.067 (2)	0.086 (2)	0.0386 (17)	−0.005 (2)	0.0029 (17)	0.0158 (16)
C27	0.070 (2)	0.076 (2)	0.0341 (15)	0.0009 (19)	−0.0117 (16)	0.0160 (15)
C28	0.072 (2)	0.075 (2)	0.0385 (16)	0.000 (2)	−0.0092 (16)	0.0015 (16)
C29	0.061 (2)	0.063 (2)	0.056 (2)	0.0001 (18)	−0.0147 (17)	0.0156 (17)
C30	0.087 (3)	0.162 (5)	0.049 (2)	0.010 (4)	0.008 (2)	−0.016 (3)
C31	0.100 (4)	0.208 (7)	0.099 (4)	0.070 (5)	−0.027 (3)	−0.056 (4)

### Geometric parameters (Å, °)

**Table:**

O1—C2	1.347 (5)	C13—H13B	0.9700
O1—C1	1.364 (4)	C14—H14A	0.9700
O2—C6	1.342 (4)	C14—H14B	0.9700
O2—C5	1.463 (4)	C15—C16	1.528 (5)
O3—C6	1.200 (4)	C15—C29	1.531 (5)
O4—C29	1.214 (4)	C15—C17	1.578 (4)
O5—C19	1.327 (3)	C16—H16A	0.9600
O5—C20	1.442 (4)	C16—H16B	0.9600
O6—C19	1.193 (3)	C16—H16C	0.9600
O7—C21	1.348 (4)	C17—C18	1.538 (4)
O7—C18	1.443 (3)	C17—C23	1.554 (4)
O8—C21	1.198 (4)	C17—H17	0.9800
O9—C30	1.345 (5)	C18—C19	1.517 (4)
O9—C26	1.445 (5)	C18—H18	0.9800
O10—C30	1.175 (7)	C20—H20A	0.9600
C1—C4	1.345 (5)	C20—H20B	0.9600
C1—H1	0.9300	C20—H20C	0.9600
C2—C3	1.330 (5)	C21—C22	1.486 (5)
C2—H2	0.9300	C22—H22A	0.9600
C3—C4	1.423 (5)	C22—H22B	0.9600
C3—H3	0.9300	C22—H22C	0.9600
C4—C5	1.494 (5)	C23—C24	1.538 (5)
C5—C9	1.535 (4)	C23—C26	1.552 (5)
C5—H5	0.9800	C23—C25	1.552 (5)
C6—C7	1.484 (5)	C24—H24A	0.9600
C7—C8	1.514 (4)	C24—H24B	0.9600
C7—H7A	0.9700	C24—H24C	0.9600
C7—H7B	0.9700	C25—H25A	0.9600
C8—C11	1.339 (4)	C25—H25B	0.9600
C8—C9	1.521 (4)	C25—H25C	0.9600
C9—C10	1.530 (4)	C26—C27	1.544 (5)
C9—C14	1.532 (4)	C26—H26	0.9800
C10—H10A	0.9600	C27—C29	1.497 (5)
C10—H10B	0.9600	C27—C28	1.549 (5)
C10—H10C	0.9600	C27—H27	0.9800
C11—C12	1.506 (4)	C28—H28A	0.9700
C11—C28	1.509 (4)	C28—H28B	0.9700
C12—C13	1.528 (4)	C30—C31	1.467 (8)
C12—C15	1.585 (4)	C31—H31A	0.9600
C12—H12	0.9800	C31—H31B	0.9600
C13—C14	1.520 (4)	C31—H31C	0.9600
C13—H13A	0.9700		
			
C2—O1—C1	105.9 (3)	H16B—C16—H16C	109.5
C6—O2—C5	119.8 (3)	C18—C17—C23	116.6 (3)
C19—O5—C20	116.7 (2)	C18—C17—C15	112.3 (2)
C21—O7—C18	116.1 (2)	C23—C17—C15	113.1 (2)
C30—O9—C26	118.8 (4)	C18—C17—H17	104.5
C4—C1—O1	111.0 (3)	C23—C17—H17	104.5
C4—C1—H1	124.5	C15—C17—H17	104.5
O1—C1—H1	124.5	O7—C18—C19	111.1 (2)
C3—C2—O1	111.0 (4)	O7—C18—C17	110.2 (2)
C3—C2—H2	124.5	C19—C18—C17	113.9 (2)
O1—C2—H2	124.5	O7—C18—H18	107.1
C2—C3—C4	107.0 (4)	C19—C18—H18	107.1
C2—C3—H3	126.5	C17—C18—H18	107.1
C4—C3—H3	126.5	O6—C19—O5	123.8 (3)
C1—C4—C3	105.1 (3)	O6—C19—C18	122.4 (3)
C1—C4—C5	126.5 (3)	O5—C19—C18	113.7 (2)
C3—C4—C5	128.4 (3)	O5—C20—H20A	109.5
O2—C5—C4	104.2 (2)	O5—C20—H20B	109.5
O2—C5—C9	111.0 (2)	H20A—C20—H20B	109.5
C4—C5—C9	116.5 (3)	O5—C20—H20C	109.5
O2—C5—H5	108.3	H20A—C20—H20C	109.5
C4—C5—H5	108.3	H20B—C20—H20C	109.5
C9—C5—H5	108.3	O8—C21—O7	122.5 (3)
O3—C6—O2	117.9 (3)	O8—C21—C22	125.7 (3)
O3—C6—C7	122.5 (3)	O7—C21—C22	111.8 (3)
O2—C6—C7	119.6 (3)	C21—C22—H22A	109.5
C6—C7—C8	117.8 (3)	C21—C22—H22B	109.5
C6—C7—H7A	107.8	H22A—C22—H22B	109.5
C8—C7—H7A	107.8	C21—C22—H22C	109.5
C6—C7—H7B	107.8	H22A—C22—H22C	109.5
C8—C7—H7B	107.8	H22B—C22—H22C	109.5
H7A—C7—H7B	107.2	C24—C23—C26	109.1 (3)
C11—C8—C7	121.9 (3)	C24—C23—C25	107.1 (3)
C11—C8—C9	124.0 (3)	C26—C23—C25	108.1 (3)
C7—C8—C9	114.0 (3)	C24—C23—C17	111.9 (3)
C8—C9—C10	110.1 (2)	C26—C23—C17	106.7 (3)
C8—C9—C14	110.3 (2)	C25—C23—C17	113.8 (3)
C10—C9—C14	110.8 (2)	C23—C24—H24A	109.5
C8—C9—C5	105.6 (2)	C23—C24—H24B	109.5
C10—C9—C5	111.5 (2)	H24A—C24—H24B	109.5
C14—C9—C5	108.3 (2)	C23—C24—H24C	109.5
C9—C10—H10A	109.5	H24A—C24—H24C	109.5
C9—C10—H10B	109.5	H24B—C24—H24C	109.5
H10A—C10—H10B	109.5	C23—C25—H25A	109.5
C9—C10—H10C	109.5	C23—C25—H25B	109.5
H10A—C10—H10C	109.5	H25A—C25—H25B	109.5
H10B—C10—H10C	109.5	C23—C25—H25C	109.5
C8—C11—C12	123.4 (3)	H25A—C25—H25C	109.5
C8—C11—C28	122.3 (3)	H25B—C25—H25C	109.5
C12—C11—C28	114.2 (3)	O9—C26—C27	110.4 (3)
C11—C12—C13	112.9 (3)	O9—C26—C23	108.8 (3)
C11—C12—C15	110.8 (2)	C27—C26—C23	114.1 (3)
C13—C12—C15	116.7 (3)	O9—C26—H26	107.8
C11—C12—H12	105.1	C27—C26—H26	107.8
C13—C12—H12	105.1	C23—C26—H26	107.8
C15—C12—H12	105.1	C29—C27—C26	111.3 (3)
C14—C13—C12	113.8 (3)	C29—C27—C28	104.4 (3)
C14—C13—H13A	108.8	C26—C27—C28	116.3 (3)
C12—C13—H13A	108.8	C29—C27—H27	108.2
C14—C13—H13B	108.8	C26—C27—H27	108.2
C12—C13—H13B	108.8	C28—C27—H27	108.2
H13A—C13—H13B	107.7	C11—C28—C27	113.5 (3)
C13—C14—C9	111.9 (2)	C11—C28—H28A	108.9
C13—C14—H14A	109.2	C27—C28—H28A	108.9
C9—C14—H14A	109.2	C11—C28—H28B	108.9
C13—C14—H14B	109.2	C27—C28—H28B	108.9
C9—C14—H14B	109.2	H28A—C28—H28B	107.7
H14A—C14—H14B	107.9	O4—C29—C27	122.3 (3)
C16—C15—C29	108.3 (3)	O4—C29—C15	123.0 (3)
C16—C15—C17	115.7 (3)	C27—C29—C15	114.0 (3)
C29—C15—C17	109.6 (3)	O10—C30—O9	121.8 (6)
C16—C15—C12	110.5 (3)	O10—C30—C31	126.1 (5)
C29—C15—C12	100.6 (3)	O9—C30—C31	112.1 (5)
C17—C15—C12	111.0 (2)	C30—C31—H31A	109.5
C15—C16—H16A	109.5	C30—C31—H31B	109.5
C15—C16—H16B	109.5	H31A—C31—H31B	109.5
H16A—C16—H16B	109.5	C30—C31—H31C	109.5
C15—C16—H16C	109.5	H31A—C31—H31C	109.5
H16A—C16—H16C	109.5	H31B—C31—H31C	109.5
			
C2—O1—C1—C4	0.0 (4)	C12—C15—C17—C18	100.0 (3)
C1—O1—C2—C3	0.0 (5)	C16—C15—C17—C23	107.4 (3)
O1—C2—C3—C4	0.0 (5)	C29—C15—C17—C23	−15.4 (4)
O1—C1—C4—C3	0.0 (4)	C12—C15—C17—C23	−125.6 (3)
O1—C1—C4—C5	179.0 (3)	C21—O7—C18—C19	82.4 (3)
C2—C3—C4—C1	0.0 (4)	C21—O7—C18—C17	−150.4 (3)
C2—C3—C4—C5	−179.0 (3)	C23—C17—C18—O7	−52.8 (3)
C6—O2—C5—C4	−166.2 (3)	C15—C17—C18—O7	80.0 (3)
C6—O2—C5—C9	−40.0 (4)	C23—C17—C18—C19	72.9 (3)
C1—C4—C5—O2	−134.2 (3)	C15—C17—C18—C19	−154.4 (2)
C3—C4—C5—O2	44.6 (4)	C20—O5—C19—O6	−2.2 (4)
C1—C4—C5—C9	103.2 (4)	C20—O5—C19—C18	179.5 (3)
C3—C4—C5—C9	−78.0 (4)	O7—C18—C19—O6	179.6 (3)
C5—O2—C6—O3	178.5 (3)	C17—C18—C19—O6	54.4 (4)
C5—O2—C6—C7	−2.7 (5)	O7—C18—C19—O5	−2.1 (3)
O3—C6—C7—C8	−162.5 (4)	C17—C18—C19—O5	−127.3 (3)
O2—C6—C7—C8	18.8 (5)	C18—O7—C21—O8	−1.8 (5)
C6—C7—C8—C11	−171.5 (3)	C18—O7—C21—C22	177.7 (3)
C6—C7—C8—C9	9.4 (5)	C18—C17—C23—C24	−47.1 (4)
C11—C8—C9—C10	−106.3 (3)	C15—C17—C23—C24	−179.4 (3)
C7—C8—C9—C10	72.8 (4)	C18—C17—C23—C26	−166.3 (3)
C11—C8—C9—C14	16.4 (4)	C15—C17—C23—C26	61.3 (4)
C7—C8—C9—C14	−164.5 (3)	C18—C17—C23—C25	74.5 (4)
C11—C8—C9—C5	133.3 (3)	C15—C17—C23—C25	−57.9 (4)
C7—C8—C9—C5	−47.7 (3)	C30—O9—C26—C27	−119.0 (4)
O2—C5—C9—C8	63.6 (3)	C30—O9—C26—C23	115.2 (4)
C4—C5—C9—C8	−177.4 (3)	C24—C23—C26—O9	−45.9 (4)
O2—C5—C9—C10	−55.9 (3)	C25—C23—C26—O9	−162.1 (3)
C4—C5—C9—C10	63.0 (3)	C17—C23—C26—O9	75.1 (3)
O2—C5—C9—C14	−178.1 (2)	C24—C23—C26—C27	−169.6 (3)
C4—C5—C9—C14	−59.2 (3)	C25—C23—C26—C27	74.2 (4)
C7—C8—C11—C12	−173.2 (3)	C17—C23—C26—C27	−48.6 (4)
C9—C8—C11—C12	5.9 (5)	O9—C26—C27—C29	−130.9 (3)
C7—C8—C11—C28	2.8 (5)	C23—C26—C27—C29	−8.0 (4)
C9—C8—C11—C28	−178.1 (3)	O9—C26—C27—C28	−11.4 (4)
C8—C11—C12—C13	2.8 (4)	C23—C26—C27—C28	111.4 (4)
C28—C11—C12—C13	−173.5 (3)	C8—C11—C28—C27	135.0 (3)
C8—C11—C12—C15	−130.2 (3)	C12—C11—C28—C27	−48.6 (4)
C28—C11—C12—C15	53.5 (3)	C29—C27—C28—C11	51.4 (4)
C11—C12—C13—C14	−33.9 (4)	C26—C27—C28—C11	−71.7 (4)
C15—C12—C13—C14	96.2 (3)	C26—C27—C29—O4	−130.9 (4)
C12—C13—C14—C9	57.0 (3)	C28—C27—C29—O4	102.9 (4)
C8—C9—C14—C13	−46.3 (3)	C26—C27—C29—C15	58.6 (4)
C10—C9—C14—C13	76.0 (3)	C28—C27—C29—C15	−67.7 (4)
C5—C9—C14—C13	−161.5 (2)	C16—C15—C29—O4	17.0 (5)
C11—C12—C15—C16	−174.1 (2)	C17—C15—C29—O4	144.1 (3)
C13—C12—C15—C16	54.8 (3)	C12—C15—C29—O4	−98.9 (4)
C11—C12—C15—C29	−59.9 (3)	C16—C15—C29—C27	−172.5 (3)
C13—C12—C15—C29	169.0 (3)	C17—C15—C29—C27	−45.4 (4)
C11—C12—C15—C17	56.0 (3)	C12—C15—C29—C27	71.6 (3)
C13—C12—C15—C17	−75.1 (3)	C26—O9—C30—O10	4.9 (7)
C16—C15—C17—C18	−27.1 (4)	C26—O9—C30—C31	−174.6 (4)
C29—C15—C17—C18	−149.8 (3)		
